# Identification of candidate genes for drought tolerance in coffee by high-throughput sequencing in the shoot apex of different *Coffea arabica* cultivars

**DOI:** 10.1186/s12870-016-0777-5

**Published:** 2016-04-19

**Authors:** Luciana Souto Mofatto, Fernanda de Araújo Carneiro, Natalia Gomes Vieira, Karoline Estefani Duarte, Ramon Oliveira Vidal, Jean Carlos Alekcevetch, Michelle Guitton Cotta, Jean-Luc Verdeil, Fabienne Lapeyre-Montes, Marc Lartaud, Thierry Leroy, Fabien De Bellis, David Pot, Gustavo Costa Rodrigues, Marcelo Falsarella Carazzolle, Gonçalo Amarante Guimarães Pereira, Alan Carvalho Andrade, Pierre Marraccini

**Affiliations:** Laboratório de Genômica e Expressão (LGE), Departamento de Genética e Evolução, Instituto de Biologia/UNICAMP, Cidade Universitária Zeferino Vaz, 13083-970 Campinas, SP Brazil; Embrapa Recursos Genéticos e Biotecnologia (LGM-NTBio), Parque Estação Biológica, CP 02372, 70770-917, Brasilia, DF Brazil; CIRAD UMR AGAP, F-34398 Montpellier, France; Embrapa Informática Agropecuária, UNICAMP, Av. André Tosello n° 209, CP 6041, 13083-886 Campinas, SP Brazil; present address: Embrapa Café, INOVACAFÉ, Campus UFLA, 37200-000 Lavras, MG Brazil

**Keywords:** Candidate gene, Coffee, Drought, Differential gene expression, RNA-Seq, Real-time PCR (RT-qPCR)

## Abstract

**Background:**

Drought is a widespread limiting factor in coffee plants. It affects plant development, fruit production, bean development and consequently beverage quality. Genetic diversity for drought tolerance exists within the coffee genus. However, the molecular mechanisms underlying the adaptation of coffee plants to drought are largely unknown. In this study, we compared the molecular responses to drought in two commercial cultivars (IAPAR59, drought-tolerant and Rubi, drought-susceptible) of *Coffea arabica* grown in the field under control (irrigation) and drought conditions using the pyrosequencing of RNA extracted from shoot apices and analysing the expression of 38 candidate genes.

**Results:**

Pyrosequencing from shoot apices generated a total of 34.7 Mbp and 535,544 reads enabling the identification of 43,087 clusters (41,512 contigs and 1,575 singletons). These data included 17,719 clusters (16,238 contigs and 1,575 singletons) exclusively from 454 sequencing reads, along with 25,368 hybrid clusters assembled with 454 sequences. The comparison of DNA libraries identified new candidate genes (*n* = 20) presenting differential expression between IAPAR59 and Rubi and/or drought conditions. Their expression was monitored in plagiotropic buds, together with those of other (*n* = 18) candidates genes. Under drought conditions, up-regulated expression was observed in IAPAR59 but not in Rubi for *CaSTK1* (protein kinase), *CaSAMT1* (SAM-dependent methyltransferase), *CaSLP1* (plant development) and *CaMAS1* (ABA biosynthesis). Interestingly, the expression of lipid-transfer protein (nsLTP) genes was also highly up-regulated under drought conditions in IAPAR59. This may have been related to the thicker cuticle observed on the abaxial leaf surface in IAPAR59 compared to Rubi.

**Conclusions:**

The full transcriptome assembly of *C. arabica*, followed by functional annotation, enabled us to identify differentially expressed genes related to drought conditions. Using these data, candidate genes were selected and their differential expression profiles were confirmed by qPCR experiments in plagiotropic buds of IAPAR59 and Rubi under drought conditions. As regards the genes up-regulated under drought conditions, specifically in the drought-tolerant IAPAR59, several corresponded to orphan genes but also to genes coding proteins involved in signal transduction pathways, as well as ABA and lipid metabolism, for example. The identification of these genes should help advance our understanding of the genetic determinism of drought tolerance in coffee.

**Electronic supplementary material:**

The online version of this article (doi:10.1186/s12870-016-0777-5) contains supplementary material, which is available to authorized users.

## Background

Coffee is the single most important tropical commodity traded worldwide and is a source of income for many developing countries in Tropics [1]. In the coffee genus, *Coffea arabica* accounts for approximately 70 % of total production worldwide, estimated at 8.5 million tons in 2015 [2]. Coffee production is subject to regular fluctuations mainly due to the natural biennial cycle but also caused by adverse climatic effects. Among them, drought is a widespread limiting factor and affects flowering and bean development, hence coffee yield [3]. Marked variations in rainfall also increase bean defects and modify the biochemical composition of beans, hence the final quality of the beverage [4]. Periods of drought may become more pronounced as a consequence of global climate change and geographical coffee growing regions may shift considerably, leading to environmental, economic and social problems [5]. In such a context, the creation of drought-tolerant coffee varieties has now become a priority for coffee research.

Genetic variability for drought tolerance exits in the coffee genus, particularly in *Coffea canephora* [6, 7] but also in *C. arabica* [8]. Although molecular mechanisms of drought tolerance have been widely studied in model plants [9], they are less well understood in *Coffee sp*. In a previous study analysing the effects of drought on gene expression, we recently identified a set of 30 genes differentially expressed in the leaves of drought-tolerant and drought-susceptible clones of *C. canephora* grown in the greenhouse under control (unstressed) and drought conditions [10, 11]. In that case, the expression of genes encoding glycine-rich proteins, heat shock proteins, dehydrins, ascorbate peroxidase, as well as trans-acting factors (such as DREB1D), for example, increased under drought conditions.

In *Coffea sp.*, EST resources have been developed for various species and tissues including roots, leaves, and fruits [12–16]. However, no genomic resources are available for shoot apices, which are considered as key organs for plant development by integrating several signals, such as environmental stimuli as well as hormones (abscisic acid [ABA], auxins, cytokinins) and transcription [17]. On the other hand, next-generation sequencing (NGS) provides new opportunities to study transcriptomic responses and to combine high-throughput sequencing with the functional annotation capacity of generated ESTs [18].

In order to identify candidate genes involved in drought tolerance in coffee plants, we collected the shoot apices from drought-tolerant IAPAR59 and drought-susceptible Rubi cultivars of *C. arabica* under control and drought conditions to generate libraries that were sequenced using the GS-FLX Titanium strategy. A reference full transcriptome was annotated and compared to pre-identify genes differentially expressed between cultivars and drought conditions. The transcription profiles of these genes were further analysed by qPCR in the plagiotropic buds of these plants.

## Methods

### Plant material

We compared two cultivars of *Coffea arabica*, the drought-susceptible (D^S^) Rubi MG1192 (also referred to hereafter as RUB) and the drought-tolerant (D^T^) IAPAR59 (also referred to hereafter as I59). Rubi did not undergo recent introgression with *C. canephora* genomic DNA, while IAPAR59 is the result of a cross between the Timor hybrid HT832/2 and the Villa Sarchi cultivar [19].

### Field experiment

Seeds of these two commercial cultivars came from fruits harvested in May 2007 in the coffee experimental fields of the Institute for Research and Rural Assistance (Incaper, Vitoria, Espirito Santo, Brazil) and germinated (September 2007) in greenhouse of this institute. Five-month-old plantlets of the Rubi and IAPAR59 were then planted (January 2008) in a field experiment (0.7 m spacing between plants and 3 m spacing between rows) at the Cerrado Agricultural Research Center (Planaltina-DF, Brazil 15°35’44”S - 47°43’52”W) under full-sunlight conditions in two blocks of 30 plants for each cultivar. Under the conditions of the Cerrado climate [20], the rainfall pattern is divided into a dry season (from May to September) followed by a wet season (from October to April) that concentrates more than 80 % of annual precipitations. For each cultivar, one control (C) block was irrigated while the drought (D) block was not irrigated during the dry seasons. For the control condition, irrigation was supplied by sprinklers (1.5 m in height) set up in the field in such a way that irrigation was uniform. Soil water content was monitored using PR2 profile probes (Delta-T Devices Ltd), and irrigation was applied regularly so as to maintain a moisture content above 0.27 cm^3^ H_2_O.cm^-1^.

### Sampling

**Fig. 1:**
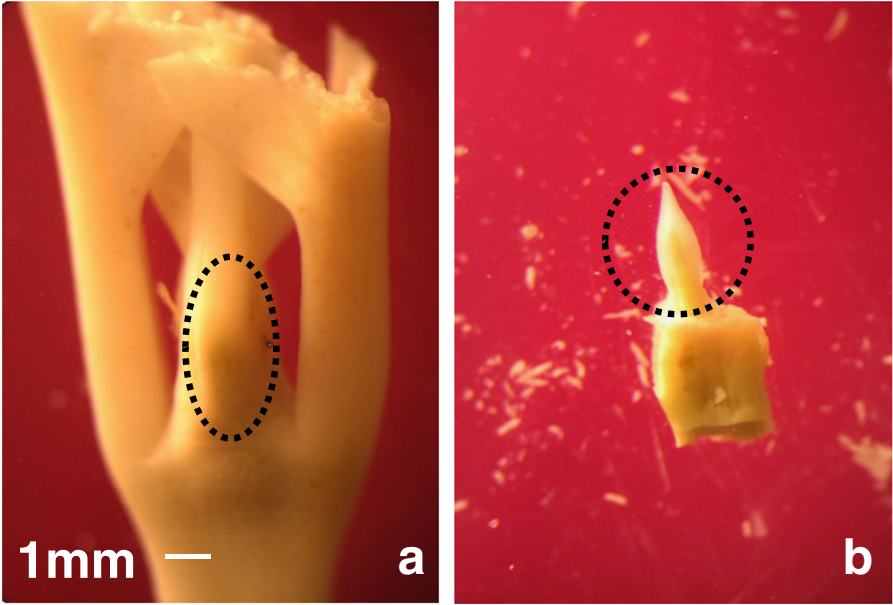
Tissue dissection of plagiotropic buds. **a** The plagiotropic buds (including small leaves) were collected from plants during the 2011 dry season and used to extract RNA for qPCR expression analysis. **b** Meristem and leaf primordium dissected from plagiotropic buds harvested during the 2009 dry season and used to extract RNA for pyrosequencing. The dotted circles show the position of meristem and leaf primordium. The same scale (white bar = 1 mm) is used for both documents

For both cultivars and experiments, leaf predawn water potentials (*Ψ*_pd_) were measured once a week during the 2009 dry season (from May to October) of (23-month-old plants) and only once in 2011 (at the end of the dry season) (47-month-old plants) using a Scholander-type pressure chamber (Plant Water Status Console, Model 3000 F01, Soil Moisture Equipment Corp, Santa Barbara, CA USA) in fully expanded leaves (8–15 cm long) from the third pair from the apex of plagiotropic branches located in the upper third of the plant canopy. For 454 sequencing, between 30 and 50 shoot apices were collected (between 10:00 and 11:00 am) from three different plants at the end of the dry season from Rubi and IAPAR59 under the control and drought conditions, and further dissected to isolate the shoot apex (Fig. 1b). For microscopic analyses, leaves identical to those used for *Ψ*_pd_ measurements were also collected from the same plants. At the end of the 2011 dry season, *Ψ*_pd_ were measured once for Rubi and IAPAR59 plants under control and drought treatments, and shoot apices were collected (Fig. 1a) for gene expression analyses (qPCR).

### RNA isolation, DNA synthesis and 454-sequencing

The plagiotropic buds were incubated for 5 min in the washing buffer (66 % chloroform, 33 % methanol, 1 % HCl) [21] and further incubated twice for 30 min under a vacuum in the fixation buffer (25 % acetic acid, 75 % ethanol RNAse-free) then cooled to 4 °C. Samples were stored in 75 % RNAse-free ethanol. For the control and drought conditions, shoot apices (meristems and primordium leaves) of three different plants were separated from plagiotropic buds under a binocular microscope by dissection and then ground to powder in liquid nitrogen using a pestle and mortar. Total RNA was extracted using the Nucleospin RNA Plant kit (Macherey-Nagel), including a DNAse-I treatment. The quality and quantity of RNA were checked with a Bioanalyzer (2100, RNA Nano 6000 Agilent). The 1^st^ strand cDNA synthesis was performed using 1 μg total RNA and the SMARTer™ PCR cDNA Synthesis Kit (Clontech). Double-stranded DNA was then produced for each library (I59-C, I59-D, RUB-C and RUB-D). For each sample, DNA (around 5 μg) was nebulized to a mean fragment size of 650 bp, ligated to an adapter using standard procedures [22] and then sequenced by performing two runs (1 library per DNA sample x 2) using GS-FLX Titanium (Beckman Coulter Genomics SA, Grenoble, France) which generated one million reads corresponding to more than 255 Mb.

### Transcriptome assembly and automatic annotation

All 454-sequencing reads were inspected for low quality reads and 454 adapters that were identified by SSAHA2 software [23]. A reference full transcriptome was then built using *C. arabica* reads originating from the present project and from the Brazilian Coffee Genome Project (BCGP) available in the GenBank public database [14, 24]. The Sanger and 454 reads were submitted for a trimming pipeline using *bdtrimmer* software [25] that was used to exclude ribosomal, vector, low quality (regions with a PHRED score less than 20) and short sequences (less than 100 bp). All sequences (454 and Sanger reads) were assembled using MIRA software [26]. The contigs formed by only Sanger reads were discarded from the full transcriptome assembly. The reference full transcriptome was annotated by Blast2GO software version 2.8 [27] using Non-Redundant protein (NCBI/NR), InterPro and Gene Ontology (GO) databases. The same program was also used to group datasets in GO according to the biological process. Further details on the automatic annotation of all contigs are provided in Additional file 1: Table S1. The complete bioinformatic pipeline used for this work is described in Additional file 2: Figure S1.

### Digital gene expression analysis

The reference full transcriptome was also used to count all 454 reads/libraries individually by parsing the ACE file generated by MIRA software. The number of sequences anchored in each contig (read counts) was subjected to differential expression analysis between the libraries using DEseq [28] and EdgeR [29] software in the R/Bioconductor package. A unigene was considered as differentially expressed when it was identified in at least one software considering fold-change ≥ 2 (or fold-change ≤ -2) and *p*-value ≤ 0.05. The libraries were compared based on (1) differentially expressed genes in IAPAR59 between C (control) and D (drought) conditions (with the calculation of fold-change based on the I59-D/I59-C ratio), (2) differentially expressed genes in Rubi between C and D conditions (RUB-D/RUB-C), (3) differentially expressed genes in the control library between Rubi and IAPAR59 (RUB-C/I59-C) and (4) differentially expressed genes in the drought library between Rubi and IAPAR59 (RUB-D/I59-D). Further information about differentially expressed genes in all the libraries is given in Additional file 3: Table S2.

### Functional annotation of differentially expressed genes

The lists of differentially expressed genes in each analysis were separated into UP and DOWN regulated and subjected to GO enrichment analysis to identify significantly enriched GO slim terms (Plant GO slim) using Blast2GO software and a *p*-value ≤ 0.05.

### Selection of candidate genes

The comparison of DNA libraries led to the identification of 80 (20 for each library) candidate genes (CGs) that were up- and down-regulated (see Additional file 3: Table S2). For each CG, primer pairs were designed using Primer Express software (Applied Biosystems) and tested of their specificity and efficiency against a mix of ss-DNAs of plagiotropic buds (data not shown). The best primer pairs (*n* = 20) were used to monitor the expression of corresponding CGs in plagiotropic buds of Rubi and IAPAR59 under control and drought conditions. These genes corresponded to *CaAEP1*, *CaCAB2*, *CaCHI1*, *CaCHI2*, *CaCHI3*, *CaDLP1*, *CaELIP3*, *CaGAS2*, *CaGRP2*, *CaH2A*, *CaHSP3*, *CaIPS1*, *CaJAMT1*, *CaMAS1*, *CaPP2*, *CaPSBB*, *CaSAMT1*, *CaSDC1*, *CaSLP1* and *CaSTK1* (Table 1). This list of CGs was increased by adding other genes such as 14 orphan genes (*CaUNK2-CaUNK7*, *CaUNK9* and *CaUNK11*-*CaUNK17* already described to present differential gene expression profiles in different organs of *C. canephora* [30]. This list was finally completed by including the *CaUNK1*, *CaUNK8* and *CaUNK10* orphan genes, and *LTP* genes that were already studied in *C. canephora* [10, 11, 31] and *C. arabica* [32], respectively.

**Table 1:** Candidate genes and corresponding primers used for qPCR experiments

Gene	Protein name	*C. canephora*	GB	ATP	SGN	Primer	Primer sequences	bp
*CaUBQ10*	Ubiquitin	Cc02_g31600	GW488515	32782	U637098	BUBI-F BUBI-R	5’ AAGACAGCTTCAACAGAGTACAGCAT 3’ 5’ GGCAGGACCTTGGCTGACTATA 3’	104
*CaAEP1* ^a^	Putative aldose 1-epimerase	Cc07_g03170	GT005185	716	U637659	716-1 F 716-1R	5' CGGTGATGTCCTCTCTGATGAG 3’ 5’ GTTGGGATGAGCTGGTTGTTC 3’	75
*CaCAB2* ^a^	Chlorophyll a/b-binding protein	Cc09_g09030	GT003492	33540	U629601	48565-F 48565-R	5’ GTTCAAGGCTGGATCCCAAA 3’ 5’ GCAAGCCCAGATAGCCAAGA 3’	100
*CaCHI1* ^a^	Class III chitinase	Cc11_g00410	GT012279	32745	U637166	50103-F 50103-R	5’ AATCAAGCGACCGTCCATTC 3’ 5’ GTGTTTCCGCTGTGGATGTG 3’	70
*CaCHI2* ^a^	Putative chitinase	Cc00_g14300	GT011845	32737	U638035	53058-F 53058-R	5’ CCTGCTCGCGGTTTCTACAC 3’ 5’ TTGTTCCAAAAGCCCCATTG 3’	70
*CaCHI3* ^a^	Chitinase-like protein	Cc03_g13720	GW491433	32875	U645893	23638-F 23638-R	5’ AAACGGCCCGTCCAGAA 3’ 5’ GCTTTGTCCTGCTGGTCCAT 3’	130
*CaDLP1* ^a^	Dirigent-like protein	Cc00_g27410	GW477731	35149	nf	39577-F 39577-R	5’ TTGGTAGTCCGGCGAGAGAA 3’ 5’ GCATATCCCCGAGCAAACCT 3’	70
*CaELIP3* ^a^	Early light-induced protein (ELIP)	Cc03_g04300	GR985685	32771	U631550	32771-F 32771-R	5’ TCGGTTGCCATGCAATCTT 3’ 5’ GCAGATGAAGCCCACAGCTT 3’	100
*CaGAS2* ^a^	Glucosyltransferase arbutin synthase	Cc02_g39100	GT697284	3945	U632419	632419-F 632419-R	5’ GCTGACGACGTTAGGATTGAGA 3’ 5’ AACTTGGCGGTGTCAACCAA 3’	101
*CaGRP2* ^a^	Glycin-rich protein	Cc00_g16260	GW430980	32799	U635030	53139-1 F 53139-1R	5’ CACATATGCTGGTGAGCCAAA 3’ 5’ AGGCATTTAAGCGCCATGAT 3’	100
*CaH2A* ^a^	Putative histone H2A	Cc01_g12440	GT723387	33557	U630412	53417-F 53417-R	5’ GCACTGGAGCTCCGGTCTAC 3’ 5’ AGCAGCATTTCCAGCCAATT 3’	80
*CaHSP3* ^a^	Heat schock protein (HSP) 70 kDa	Cc02_g08040	GR982512	33197	U636531	33197-1 F 33197-1R	5’ GGCGTCTGGCAACACGAT 3’ 5’ CGATGAGACGCTCGGTGTCT 3’	100
*CaIPS1* ^a^	Myo-inositol 1-phosphate synthase	Cc07_g15530	GT003538	10496	U632517	10496-1 F 10496-1R	5’ AAGCAACCTGAATTTGGCTGAT 3’ 5’ GAGAGGGACCATGGATTCCA 3’	100
*CaJAMT1* ^a^	Jasmonate O-methyltransferase	Cc03_g07330	GR989151	33008	U631389	47327-F 47327-R	5’ CTGTGGCTGAACCCTTGCTT 3’ 5’ TCTTTGGACATGCGATCAGAAA 3’	100
*CaMAS1* ^a^	Momilactone-A synthase	Cc00_g13640	GW479615	33413	nf	33413-F 33413-R	5’ GGGCAGAGGCACGAAAAA 3’ 5’ GGTACCCTGCCGCAACTATG 3’	60
*CaPP2* ^a^	Putative phloem protein 2 (PP2)	Cc03_g13000	GR995691	33207	U633544	33207-F 33207-R	5’ GGTGTTGGCGATGTCGAGAT 3’ 5’ TTCCTTGGGTCGAAGCTCAA 3’	90
*CaPSBB* ^a^	Photosystem II CP47 (psbB)-like protein	nf	GW447378	22102	U630312	55586-F 55586-R	5’ ATCGGAAATAATCCGGCAAA 3’ 5’ AACCATCCAATCGCTATTCCA 3’	80
*CaSAMT1* ^a^	S-adenosyl-methionine-methyltransferase	Cc03_g05630	DV672716	754	U629783	34318-F 34318-R	5’ AACGTTTGGGTGATGAATGTTG 3’ 5’ GTGCCAATAAGCCCTCTATCGT 3’	80
*CaSDC1* ^a^	S-adenosyl-L-methionine decarboxylase	Cc11_g11130	GT002431	8508	U629687	8508-1 F 8508-1R	5’ CTCGATTCCTCCCATCCTGAA 3’ 5’ TGACTGTGCCCCAGGGAATA 3’	100
*CaSLP1* ^a^	Subtilisin-like protein	Cc00_g19100	GW430663	1620	nf	7961-F 7961-R	5’ CCATCGTTCTCGGTGGTCTT 3’ 5’ GCATTGCTCCCCACATTCTT 3’	80
*CaSTK1* ^a^	Hypothetical S/T protein kinase	Cc00_g18670	GT687049	6301	U631794	6301-1 F 6301-1R	5’ CCACCCACAAGCTGTATTCTCA 3’ 5’ GACCCAATGGGATGTCATCAC 3’	80
*CaUNK1* ^c^	Unknown protein 1	Cc03_g08880	DV689820	33062	U614843	182052-F 182052-R	5’ TATAGTGTTTATGGTGTGGCTTTCAGT 3’ 5’ GTACCACCGTAGGGAGACGTATG 3’	79
*CaUNK2* ^b^	Unknown protein 2	Cc07_g01940	DV708962	31492	U637447	33353-F 33353-R	5’ GAACTTACAAACGCGCGTAACC 3’ 5’ CATGGTCGAATCCAGATTTCATT 3’	80
*CaUNK3* ^b^	Unknown protein 3	nf	nf	22823	nf	22823-F 22823-R	5’ GGAAGCATGCACACAGAAAATAGA 3’ 5’ TTCCTGTTTACGTCTTTTTCAATTGA 3’	80
*CaUNK4* ^b^	Unknown protein 4	Cc06_g11210	GW465088	39984	nf	55677-F 55677-R	5’ GCTGTGGTTTTAAAGTTTTGATGGA 3’ 5’ TGCAAAATTAAGGTCCCAACAGT 3’	81
*CaUNK5* ^b^	Unknown protein 5	Cc08_g09510	GW474926	4578	nf	4578-F 4578-R	5’ GGAGTTCCTGTCCGAAGTTGTT 3’ 5’ GGCATGCTGTCACCTGAAAA 3’	80
*CaUNK6* ^b^	Unknown protein 6	Cc03_g06850	GT002178	34993	U632634	34993-F 34993-R	5’ AAGCCAATGCCGATCGATT 3’ 5’ CGCCGCCGAAGATCTCTAG 3’	100
*CaUNK7* ^b^	Unknown protein 7	Cc03_g00560	GW444736	33613	U631416	25639-F 25639-R	5’ CGAGGAAGCTGAAGGAAAGGA 3’ 5’ TCCGACTGGCCTAACAAGGT 3’	61
*CaUNK8* ^b^	Unknown protein 8	Cc00_g04970	DV695331	33190	U640780	LP18101-F LP18100-R	5’ CTCGCGTGGCCGAGATC 3’ 5’ CCCTCACATTTCCACGTGAAT 3’	100
*CaUNK9* ^b^	Unknown protein 9	Cc03_g08920	GT649500	32762	U636808	30926-F 30926-R	5’ CGGAGGAGGCCATGGAGGT 3’ 5’ CCGTGTCCATAACCACCATGT 3’	123
*CaUNK10* ^c^	Unknown protein 10	nf	GT648004	14813	U645073	D18240-F D18240-R	5’ TAGCCTTGTTCTTTTAGGGAGTCTTATC 3’ 5’ AGAGCTTCGTCCAGGAAGAAGA 3’	134
*CaUNK11* ^b^	Unknown protein 11	Cc03_g14330	GR991912	8598	U637116	32792-F 32792-R	5’ GCTGGGAAAGCTACAGAAACCA 3’ 5’ GAACTCCAACGCCAAGCATT 3’	100
*CaUNK12* ^b^	Unknown protein 12	Cc10_g12840	nf	53029	nf	53029-F 53029-R	5’ CTTCACACCATTCAGACAATCGA 3’ 5’ GACCGTAATTGGGCGTCAAT 3’	100
*CaUNK13* ^b^	Unknown protein 13	Cc00_g17760	GT673421	14198	U639484	33980-F 33980-R	5’ ATTGCCCTGTTTGCATGCAT 3’ 5’ CTGCATGGTGATTGTCCTCAGT 3’	100
*CaUNK14* ^b^	Unknown protein 14	Cc00_g16260	GT672564	48325	U635030	11524-F 11524-R	5’ GGCGGTTGTCATGGATACG 3’ 5’ TTTGGCTCACCAGCATATGTG 3’	119
*CaUNK15* ^b^	Unknown protein 15	Cc00_g04970	GR983286	33190	U636790	05517-F 05517-R	5’ AAAATTTCACCACGGCAAGCT 3’ 5’ TTGCCTCCCTCACATTTCCA 3’	72
*CaUNK16* ^b^	Unknown protein 16	nf	GW464209	9761	U639049	18112-F 18112-R	5’ TGTGAACTGCCATCCCAAGA 3’ 5’ AAGACTACCATGTCCAACAACTTCAG 3’	88
*CaUNK17* ^b^	Unknown protein 17	Cc03_g08920	GT685623	32762	U636800	42747-F 42747-R	5’ AGGTGGCTGCCAAGTCAGTT 3’ 5’ ATGGTACTTGGCTTCTCCTTCCTC 3’	71
*CaLTP1* ^d^ *CaLTP2* ^d^	Non-specific lipid transfer protein (nsLTP)	Cc11_g09700	HG008739 HG008740	46897	U632702	LTP-R2 LTP-FT	5’ CACCATTACATGGGAACGTTGC 3’ 5’ CTGTGGTCTGAAATGGCCAACT 3’	120
*CaLTP3* ^d^	Non-specific lipid transfer protein (nsLTP)	Cc04_g06890	HG008741	33368	U632702	LTP-R1 LTP-FT	5’ ATTCAACACCATTACTAGTTTTCGAGC 3’ 5’ CTGTGGTCTGAAATGGCCAACT 3’	113
*LTP* ^d^		nf		-	U632702	LTP-F100 LTP-R100	5’ TGCAATTTTATCAAAGATCCAGC 3’ 5’ AGTTGGCCATTTCAGACCACA 3’	93

Gene names were assigned based on the best BLAST hit obtained by comparing the coffee ESTs with public databases. *C. canephora* means coffee sequences that aligned with the candidate genes using BLASTx searches against NR/NCBI and filtration (http://coffee-genome.org [59]). GenBank (GB: http://blast.ncbi.nlm.nih.gov/Blast.cgi), ATP (http://www.lge.ibi.unicamp.br/cirad/) and SGN (Sol Genomics Network, http://solgenomics.net/) accession numbers of coffee ESTs are also given, as well as the length of base pairs (bp) of amplicons. nf: no-hits found (SGN: tools/blast/SGN Clusters [current version] / Coffee species Clusters, GB: BLASTn/Nucleotide collection [nr/nt]). The size of amplicons is based on the unigene. (^a^): candidate genes (*n* = 20) identified during this study. (^b^): orphan genes (*n* = 14) previously described [35] and analysed in this study. (^c^): orphan genes (*n* = 3) with expression already been studied in leaves of D^T^ and D^S^ clones of *C. canephora* conilon [10, 11, 36]. (^d^): LTP-encoding genes were previously described [37]

### Real-time quantitative PCR assays

For qPCR experiments, plagiotropic buds containing shoot apices and small leaves (Fig. 1a) were immediately frozen in liquid nitrogen after collection, and stored at -80 °C before being extracted and converted into single-strand cDNA as previously described [33]. Real-time qPCR assays were carried out using the protocol recommended for the use of 7500 Fast Real-Time PCR Systems (Applied Biosystems, Foster City, CA, USA). DNA preparations were diluted (1/50) and tested by qPCR using CG primer pairs (Table 1). RT-qPCR was performed with 1 μl of diluted ss-DNA and 0.2 μM (final concentration) of each primer in a final volume of 10 μl with SYBR green fluorochrome (SYBRGreen qPCR Mix-UDG/ROX, Invitrogen). The reaction mixture was incubated for 2 min at 50 °C (Uracil DNA-Glycosilase treatment), then for 5 min at 95 °C (inactivation of UDGase), followed by 40 amplification cycles of 3 sec at 95 °C and finally for 30 sec at 60 °C. Data were analysed using SDS 2.1 software (Applied Biosystems) to determine cycle threshold (Ct) values. The specificity of the PCR products generated for each set of primers was verified by analysing the Tm (dissociation) of amplified products. PCR efficiency (E) was estimated using absolute fluorescence data captured during the exponential phase of amplification of each reaction with the equation E (in %) = (10^(-1/slope)^ -1) x 100 [34]. Efficiency values were taken into account in all subsequent calculations. Gene expression levels were normalized to expression levels of *CaUBQ10* as a constitutive reference. Relative expression was quantified by applying the formula (1 + E)^−ΔΔCt^ where ΔCt _target_ = Ct _target gene_ – Ct _reference gene_ and ΔΔCt = ΔCt _target_ – ΔCt _internal calibrator_, with the internal reference always being the Rubi-control (RUB-C) sample with relative expression equal to 1.

### Leaf histological analysis of cuticle

Mature leaves of the IAPAR59 and Rubi genotypes were fixed for 48 h in 100 mM phosphate buffer at pH 7.2, supplemented with 1 % (v/v) glutaraldehyde, 2 % (v/v) paraformaldehyde, and 1 % (w/v) caffeine, at room temperature [35]. The samples were dehydrated and embedded in Technovit 7100 resin (Heraeus Kulzer) according to the manufacturer’s recommendations. Three-micrometer semi-thin sections were cut with glass knives on a Leica RM2065 Microtome. The resulting sections were double stained according to Buffard-Morel et al. [36]. Briefly, polysaccharides were stained dark pink with periodic acid Schiff (PAS) and soluble proteins were stained blue with naphthol blue-black (NBB) [37]. Sections were then mounted in Mowiol. The slides were observed with a Leica DM6000 microscope (Leica, Germany) under bright field or epifluorescent light (A4 filter). Pictures were taken with a Retiga 2000R camera (QImaging Co.) and the images were processed with Volocity 4.0.1 (Improvision, Lexington, MA, USA). Cuticle thickness was measured with the freeware Image J software (http://imagej.nih.gov/ij/). Experiments were conducted on the “Plate-Forme d’Histocytologie et Imagerie Cellulaire Végétale (PHIV platform)” (http://phiv.cirad.fr/) using microscopes belonging to the Montpellier Rio Imaging platform (www.mri.cnrs.fr). The results are expressed as means (μm) of 11 measured values. The data were statistically processed using (1) an analysis of variance computer program (Statistica, StatSoft, Inc.), and (2) the Student-Newman-Keuls (SNK) mean comparison test [38] when the effect of the factor tested was found to be statistically significant. A probability level of *P* ≤ 0.05 was considered significant for all the statistical analyses.

## Results

### Monitoring drought under field conditions

**Fig. 2:**
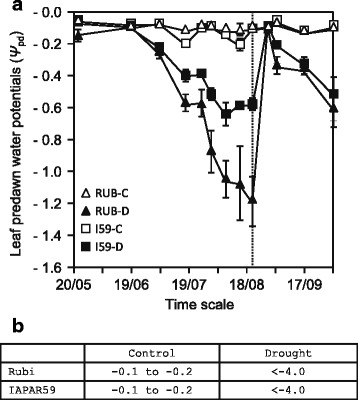
Predawn leaf water potentials (*Ψ*_pd_) measured in plants of *C. arabica*. Rubi (RUB, triangle) and IAPAR59 (I59, square) cultivars were grown under control (C, open symbols) and drought (D, black symbols) conditions. *Ψ*_pd_ values (expressed in mega-Pascal, MPa) were measured once a week during the 2009 dry season (23-month-old plants) (**a**). The time scale is in days and months (dd/mm, from 20/05 to 02/10). Vertical bars are standard deviations (*n* = 9 leaves) and the dashed vertical line (20/08) represents the harvest point of plagiotropic buds for RNA extraction for 454 sequencing and leaves for microscopic analyses. **b** *Ψ*_pd_ of Rubi and IAPAR59 plants (47-month-old plants) measured at the end of the 2011dry season. In this case, *Ψ*_pd_ values ranged from -0.1 to -0.2 MPa for the control conditions, but were below (< -4.0 MPa = severe drought) the range of use of a Scholander-type pressure chamber for drought conditions

In 2009, leaf predawn water potential (*Ψ*_pd_) values were similar in the leaves of irrigated Rubi and IAPAR59 plants, ranging from -0.06 to -0.16 MPa (Fig. 2a). This confirmed the unstressed status of these plants which were considered as the control in our experiment. At the same time, the *Ψ*_pd_ values decreased gradually during the dry season in the leaves of Rubi and IAPAR59 under drought conditions reaching the lowest values at the end of the dry season (Fig. 2a). At that time, the less negative *Ψ*_pd_ values in IAPAR59 indicated that it had better access to soil water. The first rains then occurred and the *Ψ*_pd_ values of drought-stressed plants increased almost to those measured in irrigated plants, illustrating the complete recovery of stressed plants. In 2011, *Ψ*_pd_ was measured at the peak of the drought (end of dry season). Under drought conditions, both Rubi and IAPAR59 had similar *Ψ*_pd_ values that were more negative than those measured in 2009, indicating more severe drought stress in 2011 (Fig. 2b).

### Sequencing, assembly and annotation of the *Coffee* shoot apex transcriptome

The final reference assembly generated a total of 34,743,872 bp (34.7 Mbp) with coverage of 6.5x and 43,087 clusters, corresponding to 41,512 contigs and 1,575 singletons. These data are composed of: (1) 17,719 clusters (16,238 contigs and 1,575 singletons) from 454 sequences, exclusively; and (2) 25,368 hybrid clusters that contain 454 reads, and at least one contig from Sanger sequencing (public database). The contigs formed by only Sanger reads were discarded from the full transcriptome assembly. On average, 22.4 % and 55.6 % of the total raw data were discarded from Sanger and 454, respectively, due to low quality. After removing the adapters, these reads had a size of 379.2 bp (on average). The statistical data for the Sanger and 454 reads are listed in Table 2.

**Table 2:** Characteristics of reads used in this work

Libraries	Total reads	Trimmed reads	Average length of reads
Public Sanger database	195,110	151,403	518
I59-C	135,304	66,641	325
I59-D	282,213	112,518	351
RUB-C	230,064	101,394	360
RUB-D	345,751	153,572	342
Total	1,188,442	585,528	379.2

Statistics of all reads used in this work: public Sanger reads and 454 sequenced reads from two cultivars under two conditions. Cultivars (RUB: Rubi and I59: IAPAR59) of *C. arabica* and treatments (*C* control and *D* drought) are indicated. The number of total reads, trimmed reads and average read length (in bp) are indicated

Transcriptome annotation by Blast2GO using Non-Redundant protein (NCBI/NR) and InterPro databases resulted in 36,965 transcriptome clusters (85.8 %) with a known protein function, 1,824 conserved proteins of unknown function (4.2 %), 1,515 proteins identified by InterPro only (3.5 %) and 2,783 unidentified proteins (6.5 % no-hits found).

The results of the digital gene expression analysis (Table 3) showed more differentially expressed genes (DEG) in the cultivars Rubi (RUB) and IAPAR59 (I59) cultivars under drought (D) conditions (RUB-D/I59-D), totalling 490 clusters (1.14 % of the total), with 320 clusters classified as up-regulated. Under the control (C) conditions, a few DEG were found (RUB-C/I59-C), corresponding to 184 clusters (0.43 % of total clusters). The comparison between control and drought conditions showed a prevalence of up-regulated genes (165 clusters) and a total of 226 DEG in IAPAR59 (I59-D/I59-C) with 0.52 % of total clusters, and 343 clusters in Rubi (RUB-D/RUB-C) with 0.80 % of total clusters.

**Table 3:** Reads showing differential expression between cultivars and/or treatments

Libraries	EdgeR DEG (% of total clusters)	DEseq DEG (% of total clusters)	Total DEG (% of total clusters)	Up-regulated clusters (% of total clusters)	Down-regulated clusters (% of total clusters)
I59-D/I59-C	209 (0.49 %)	176 (0.41 %)	226 (0.52 %)	165 (0.38 %)	61 (0.14 %)
RUB-D/RUB-C	323 (0.75 %)	306 (0.71 %)	343 (0.80 %)	251 (0.58 %)	92 (0.21 %)
RUB-C/I59-C	173 (0.40 %)	169 (0.39 %)	184 (0.43 %)	104 (0.24 %)	80 (0.19 %)
RUB-D/I59-D	392 (0.91 %)	433 (1.00 %)	490 (1.14 %)	320 (0.74 %)	170 (0.39 %)

Differentially expressed genes (*DEG*) were obtained with the R/Bioconductor packages DEseq and EdgeR. Total DEG values mean the union of DEseq and EdgeR results. The calculation of percentage was based on total of clusters (43,087 clusters). Cultivars (RUB Rubi and I59: IAPAR59) of *C. arabica* and treatments (*C* control and *D* drought) are indicated

**Fig. 3:**
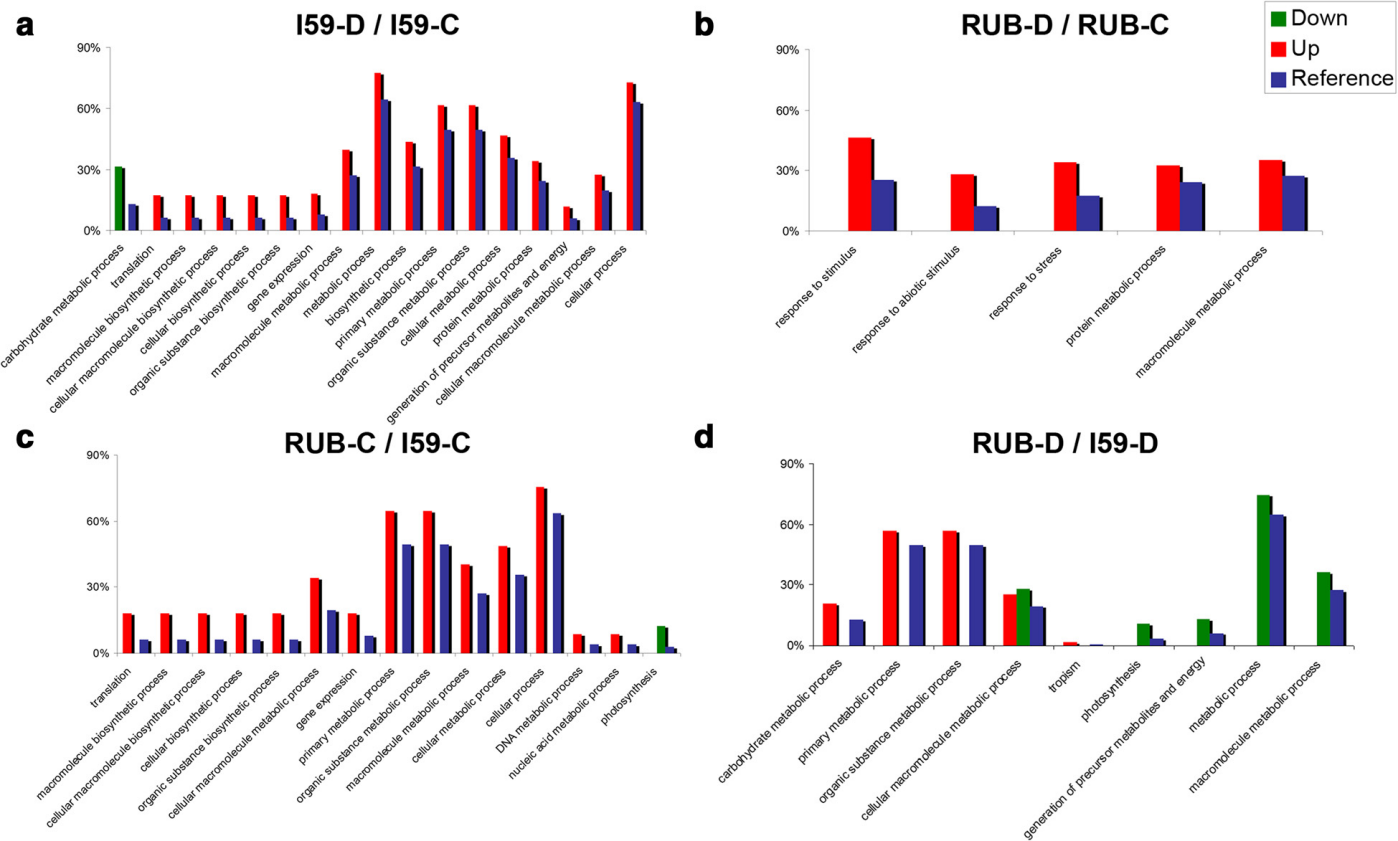
Gene ontology (GO) enrichment analysis on a list of differentially expressed genes up- and down-regulated under four conditions. The calculation of fold change was based on the ratio of: (**a**) I59-D/I59-C; (**b**) RUB-D/RUB-C; (**c**) RUB-C/I59-C; and (**d**) RUB-D/I59-D. The Y axis indicates the number of genes normalized by the total number of genes used in each comparison from each library. Cultivars (RUB: Rubi and I59: IAPAR59) of *C. arabica* and treatments (C: control and D: drought) are indicated

The results of the gene ontology (GO) enrichment analysis are shown in Fig. 3 and all GO enrichment data are listed in Additional file 1: Tables S1 and Additional file 3: Table S2. For IAPAR59, the comparison of drought and control conditions (I59-D/I59-C) identified over-represented GO terms characterized by up-regulated genes involved in expression (gALL_c3501) and translation (gALL_c2033, gALL_c4461, gALL_c6492) processes and in the generation of precursor metabolites and energy (gALL_c921, gALL_c4013, gALL_c4540). For Rubi, a comparison of the RUB-D/RUB-C libraries revealed an over-representation of the following GO terms which were up-regulated: protein metabolic process (gALL_c2021, gALL_c3355), response to stress (gALL_rep_c33197/*CaHSP3*) and response to abiotic stimulus (gALL_rep_c32771/*CaELIP3*, gALL_c2829, gALL_rep_c32766). When comparing both cultivars under drought conditions (RUB-D/I59-D), GO terms were identified related to increased enrichment of tropism for up-regulated genes (gALL_c1270, gALL_c1524, gALL_c1864) and photosynthesis for down-regulated genes (gALL_c27215, gALL_rep_c34074, gALL_rep_c34746). Under the control conditions (RUB-C/I59-C), proteins of translational machinery were identified for up-regulated genes (gALL_c3061, gALL_c16674, gALL_c19094) and photosynthesis for down-regulated genes (gALL_rep_c34074, gALL_rep_c37283, gALL_rep_c50892).

### Expression profiles of candidate genes

Among the candidate genes (CGs) identified *in silico* as presenting up- and down-regulation, expression profiles from 20 of them were analysed by qPCR together with the expression of 17 orphan genes (3 of them already studied in *C. canephora* [10, 11, 30, 31]) and *LTP* genes [32]. For all these genes, expression profiles were analysed in plagiotropic buds of Rubi and IAPAR59 under control and drought conditions. These results are presented in separate sections below, according to the observed expression patterns.

#### Genes with induced expression under drought conditions

**Fig. 4:**
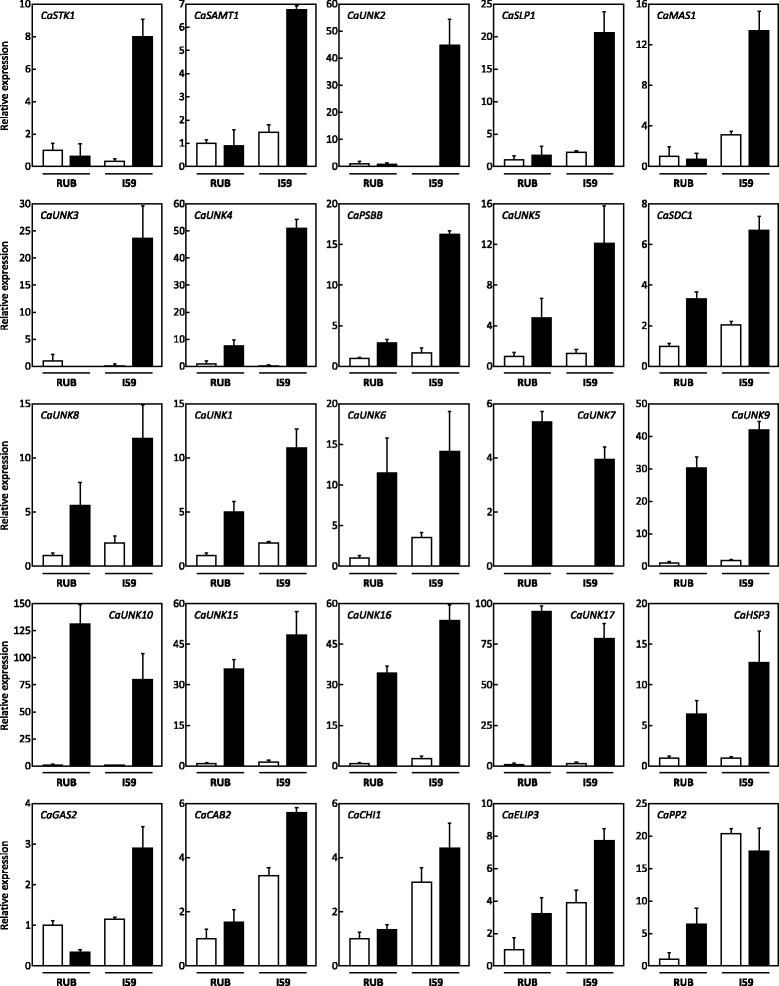
Expression profiles of genes up-regulated under drought conditions. Gene expression was analysed in plagiotropic buds of Rubi (RUB) and IAPAR59 (I59) cultivars of *C. arabica* grown under control (white isobars) and drought (black isobars) conditions. The gene names are indicated in the histograms. Transcript abundances were normalized using the expression of the *CaUBQ10* gene as the endogenous control. Results are expressed using RUB-C as the reference sample (Relative expression = 1). Values of three technical replications are presented as mean ± SD (bar)

Twenty-five genes showing up-regulated expression profiles under drought conditions, mainly in IAPAR59 and to a lesser extent in Rubi, were identified (Fig. 4). This was observed for *CaSTK1* which encodes a putative oxidative stress response serine/threonine protein kinase with 87 % identity with a predicted protein of *Populus trichocarpa* (XP_002299433). In that case, expression of this gene was highly induced by drought in the D^T^ cultivar IAPAR59. Similar profiles were also observed for the *CaSAMT1* gene encoding a putative S-adenosyl-L-methionine-dependent methyltransferase and the orphan genes *CaUNK2* and *CaUNK3*. The latter gene had no open reading frame but presented high identity (*e*-value 2E^-45^) with the SGN-U637447 contig and also with various coffee ESTs mainly found in *C. canephora* cherries at early developmental stages (data not shown).

Expression of the *CaSLP1* gene encoding a putative protein homologous (65 % identity, 74 % similarity) to a protein of *Nicotiana benthamiana* containing a peptidase S8/subtilisin-related domain, was also higher in IAPAR59 than in Rubi under drought conditions. A similar situation was observed for the *CaMAS1*gene encoding a protein of 311 amino acid residues sharing similarities (*e*-value 2E^-121^, 66 % identity, 82 %, similarity) with momilactone A synthase-like protein from *Vitis vinifera* (XP_002275768) that contains a secoisolariciresinol dehydrogenase conserved domain.

Similar expression profiles, characterized by high up-regulation under drought conditions particularly in IAPAR59, were observed for the orphan genes *CaUNK1*, *CaUNK4*, *CaUNK5*, *CaUNK8*, and for *CaPSBB* (similar to the gene of *C. arabica* chloroplast genome encoding the photosystem II CP47 chlorophyll apoprotein) and *CaSDC1* encoding a putative protein related (81 % identity, 88 %, similarity) to the adenosylmethionine decarboxylase proenzyme of *Catharanthus roseus*). Expression of the *CaUNK6* gene was also induced under drought conditions but without significant difference in expression between the two cultivars.

Interestingly, the expression profiles of orphan genes *CaUNK7*, *CaUNK9, CaUNK10*, *CaUNK15*, *CaUNK16* and *CaUNK17* were similar to that of HSP-encoding gene *CaHSP3* in the sense that gene expression was highly up-regulated under drought conditions in both cultivars. In the case of *CaUNK10,* it is worth noting that expression increased 145- and 88-fold under drought conditions in Rubi and IAPAR59, respectively.

Under drought conditions, expression of the *CaGAS2* gene encoding a putative protein homologous (73 % identity, 86 % similarity) to the arbutin synthase from *Rauvolfia serpentina* (AJ310148), was slightly increased in IAPAR59 but reduced in Rubi. The *CaCAB2*, *CaCHI1* and *CaELIP3* genes encoding a photosystem II light harvesting chlorophyll A/B binding protein of *Gardenia jasminoides* (ACN41907), a class III chitinase of *C. arabica* (ADH10372) and an early light-induced protein (ELIP) of *Glycine max* (NP_001235754), respectively, showed similar profiles but with lower expression in Rubi than in IAPAR59, under control and drought conditions. Lastly, expression of the *CaPP2* gene encoding a putative phloem protein 2 (PP2) of *Vitis vinifera* (XP_002279245) increased under drought conditions in Rubi but was quite stable in IAPAR59 under both conditions.

#### Expression of type II nsLTP genes

**Fig. 5:**
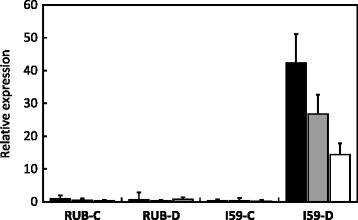
Expression of *nsLTP* genes. Expression of *CaLTP1-CaLTP2* (*CaCe*: white isobars), *CaLTP3* (*CaCc*: grey isobars) and all (*CaLTP1, CaLTP2* and *CaLTP3*: black isobars) genes was analysed by qPCR in plagiotropic buds of Rubi (RUB) and IAPAR59 (I59) cultivars of *C. arabica* grown under control (C) and drought (D) conditions, using the LTP-FT/LTP-R2, LTP-FT/LTP-R1 and LTP-F100/LTP-R100 primer pairs, respectively [37]. Expression levels are expressed in arbitrary units (AU) of *nsLTP* genes using the expression of the *CaUBQ10* gene as the endogenous control and RUB-C (with LTP100 primers) as the reference sample (Relative expression = 1). Values of three technical replications are presented as mean ± SD (bar)

The expression of Type II nsLTP-encoding genes was also monitored using the primer pairs LTP-FT/LTP-R1 (specific to the *CaLTP1* and *CaLTP2* genes from the *C. eugenioides* sub-genome of *C. arabica*, hereafter referred to as *CaCe*), LTP-FT/LTP-R2 (specific to *CaLTP3* genes from the *C. canephora* of *C. arabica*, hereafter *CaCc*) and LTP-F100/LTP-R100 recognizing all homologous genes [32]. No expression of *nsLTP* genes was detected under the control conditions in both cultivars (Fig. 5). However, expression of *nsLTP* genes was highly up-regulated in IAPAR59 but not in Rubi under drought conditions. It is worth noting that the *CaLTP1*-*CaLTP2* and *CaLTP3* genes were co-expressed in IAPAR59, and that the expression of *CaCc* genes was slightly higher than that of *CaCe* genes.

#### Drought influences leaf cuticle thickness

**Fig. 6:**
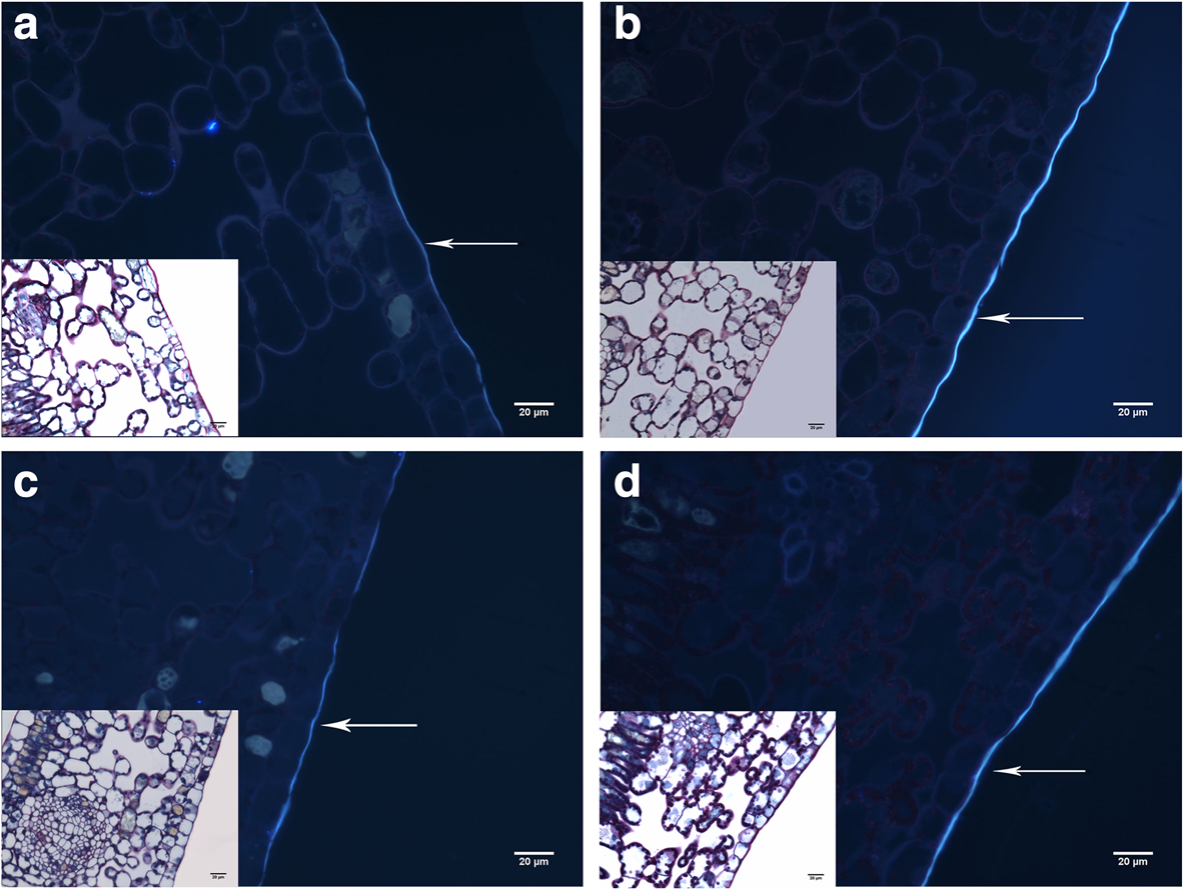
Comparative analysis of leaf histological cross sections of IAPAR59 (**a** and **b**) and Rubi (**c** and **d**) cultivars of *C. arabica* under control (irrigation: **a** and **c**) and drought (**b** and **d**) conditions. Samples were double stained with Schiff and NBB and observed under wide field (at the bottom left of each image) and fluorescent microscopy (A4 filter). LE = Lower (abaxial) epidermis. The white arrows indicate the fluorescent cuticle. Values of leaf cuticle thickness are given in Table 4. Bars = 20 μm

Leaf anatomical analyses were also performed, revealing that the abaxial epidermis of IAPAR59 had a thicker cuticle than Rubi under drought conditions (Fig. 6). There was also a strong interaction between genotype and drought conditions (F1, 40 = 16,2). For example, in the D^T^ cultivar IAPAR59, the abaxial epidermis cuticle thickness greatly increased under drought conditions compared with the control treatment (Table 4). However, no significant variation in abaxial epidermis cuticle thickness could be observed between the control and drought treatments for Rubi leaves.

**Table 4:** Influence of drought on leaf cuticle thickness

	Cuticle thickness (μm)	
Treatment	IAPAR59	Rubi
Control	1.49 ± 0.19^(a)^	1.75 ± 0.15^(b)^
Drought	1.98 ± 0.19^(c)^	1.73 ± 0.28^(b)^

Leaves of IAPAR59 and Rubi cultivars of *C. arabica* grown under control (irrigation) and drought conditions were analysed to measure the cuticle thickness of the abaxial faces. Values (in μm) correspond to the average calculated from 11 independent measurements. Those marked with different letters are significantly different (Student-Newman-Keuls mean comparison test, *P* < 0.05)

#### Genes with reduced expression under drought conditions

**Fig. 7:**
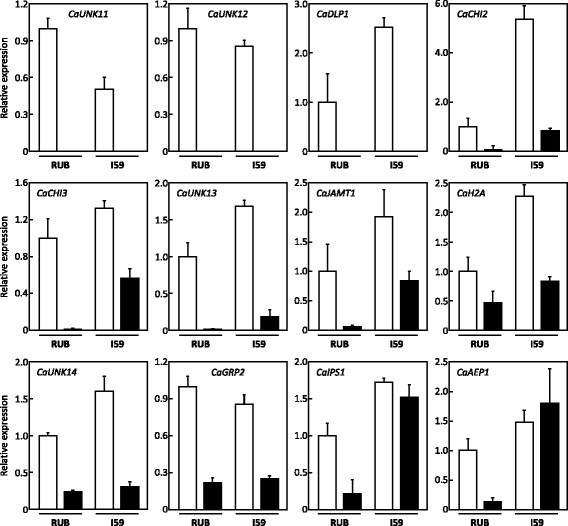
Expression profiles of genes down-regulated under drought conditions. Gene expression was analysed in plagiotropic buds of Rubi (RUB) and IAPAR59 (I59) cultivars of *C. arabica* grown under control (white isobars) and drought (black isobars) conditions. The gene names are indicated in the histograms. Transcript abundances were normalized using the expression of the *CaUBQ10* gene as the endogenous control. Results are expressed using RUB-C as the reference sample (Relative expression = 1). Values of three technical replications are presented as mean ± SD (bar)

The qPCR experiments led to the identification of several genes whose expression was reduced under drought conditions (Fig. 7). In both cultivars, expression of the orphan genes *CaUNK11* and *CaUNK12*, and of the *CaDLP1* gene encoding a putative protein containing a dirigent-like protein domain homologous to the hypothetical protein (CAN61316) of *Vitis vinifera*, was greatly reduced under drought conditions. Expression of the *CaCHI2* gene encoding a protein homologous to the putative chitinase of *Catharanthus roseus* (ADK98562), was 5-fold higher in IAPAR59 than in Rubi under the control conditions but decreased under drought conditions. However, the expression level of the *CaCHI2* gene was similar in IAPAR59 and Rubi under drought conditions. For the genes *CaCHI3* (putative protein related to chitinase-like protein *Artemisia annua* [ABJ74186]), *CaUNK13* and *CaJAMT1* (putative protein containing a methyltransferase domain [pfam03492] found in enzymes acting on salicylic acid, jasmonic acid and 7-methylxanthine), similar expression profiles were found. In these cases, drought reduced gene expression in both cultivars but expression levels were always higher in IAPAR59 than in Rubi, particularly for *CaJAMT1*.

Gene expression levels of the *CaH2A* (H2A histone protein), *CaGRP2* (putative glycin-rich protein) and *CaUNK14* genes, were similar in Rubi and IAPAR59. For the *CaAEP1* (putative aldose 1-epimerase) and *CaIPS1* (myo-inositol 1-phosphate synthase) genes, gene expression remained high in IAPAR59 under both control and drought conditions, but decreased drastically in Rubi under drought conditions.

## Discussion

In this study, we obtained 34.7 Mbp (coverage 6.5x) of sequences with longer reads (mean of 379.2 bp) from plagiotropic shoot apices enriched in meristems and primordium leaves of two cultivars of *C. arabica* under control (irrigation) and drought conditions. These sequences were assembled giving 43,087 clusters (17,719 contigs exclusively from 454-sequencing and 25,368 hybrid contigs formed by 454 and Sanger sequences) with a mean size ≥ 300 bp each. These RNAseq data, which complement those already available in public databases for coffee ESTs (407 million ESTs: dbEST release June 2015), can be considered as innovative and relevant in the sense that they were produced from *C. arabica* tissues (meristems) that have never previously been studied [39].

The transcriptome annotation by Blast2GO provided information based on the nomenclature and organism of origin of genes in the NCBI/NR database, the enzyme family, a functional analysis of proteins from the InterPro database, and metabolic functions, biological processes and cellular location from gene ontology. Our results showed that a large percentage of transcriptome alignment had 36,965 hits with known function (85.8 %), 1,824 genes with unknown function (4.2 %) both in the NCBI/NR database, and only 1,515 hits in the Interpro database (3.5 %), thereby enabling the identification of most genes. With this analysis, we identified 34,857 genes related to *Coffea* sp. (80.9 % of the total). We also found 1,383 genes from *Solanum* sp., 573 genes from *Populus trichocarpa*, 482 genes from *Vitis vinifera* and 156 genes from *Arabidopsis* sp. Thus, the transcriptome was aligned with several genes from different plant species and these genes may be conserved among these species, including *Coffea* sp. On the other hand, our results also included 2,783 “no-hit” genes (6.5 %), perhaps indicating the presence of unannotated or new genes.

The comparisons of DNA libraries undertaken during this work led to the identification of 1,243 genes (Table 3: ∑ Total DEG %) with differential expression profiles *in silico* between the drought-susceptible (Rubi) and drought-tolerant (IAPAR59) cultivars of *C. arabica* with drought conditions. The expression profiles of these genes, as well as those of other previously identified genes [10, 11, 30–32], were analysed by qPCR in plagiotropic buds (containing meristems and small leaves) taken from control and drought-stressed plants of Rubi and IAPAR59. For most of the CGs identified during this work, in vivo gene expression profiles confirmed those deduced from *in silico* comparisons of DNA libraries. For example, this was the case for the *CaHSP3* (heat shock protein) gene whose up-regulated expression under drought conditions can be considered as a “molecular control” of stress applied to the plants during this study and confirmed by leaf water potential (*Ψ*_pd_) measurements. Many ESTs encoding putative HSPs were also found in leaf cDNA libraries of *C. arabica* (SH2) and *C. canephora* (SH3) plants grown under drought conditions [31], heat stress [40], leaf infection by *Hemileia vastatrix* [15, 16] and also during bean development [14].

Our results also identified several genes differentially expressed in plagiotropic buds of IAPAR59 and Rubi, as for the *CaSTK1* gene encoding a putative serine/threonine protein kinase containing a conserved domain (cd06610) of mitogen-activated protein kinases (MAPKs). These kinases are known to have a central role in the transduction of extra- and intracellular signals in plants, including cell division and differentiation, as well as in responses to various types of stress [41]. In *Pisum sativum*, there is evidence that the MAPK cascade is involved in ABA-regulated stomatal activity as well as ABA-induced gene expression in the epidermal peels [42]. In a recent study, Shen et al. [43] showed that the phosphorylation of OsWRKY30 protein by MAPKs is a key step in conferring drought tolerance in transgenic rice. According to our results, higher *CaSTK1* expression under drought conditions in IAPAR59 than in Rubi could enhance the MAPK cascade and therefore be involved in the drought tolerance of IAPAR59. In this cultivar, the over-expression of *CaSAMT1* under drought conditions is also particularly interesting because this sequence encodes a putative S-adenosyl-L-methionine-dependent methyltransferase related to the TUMOROUS SHOOT DEVELOPMENT2 (TSD2) gene. In *Arabidopsis thaliana*, *tsd2* is a pleiotropic mutation that affects leaf, root and shoot meristem development [44]. Expression of a TSD2:: GUS reporter gene has mainly been detected in meristems where this gene is essential for cell adhesion and coordinated plant development. The weaker expression of *CaSAMT1* in Rubi than in IAPAR59 under drought conditions, points to the existence of major developmental differences between these two cultivars. The differential expression in Rubi and IAPAR59 of the *CaSLP1* gene encoding a putative subtilisin-like protein is also worth noting. In *Arabidopsis*, the subtilisin-like serine-protease *SDD1* (*stomatal density* and *distribution*) gene was shown to be strongly expressed in stomatal precursor cells (meristemoids and guard mother cells) [45]. In addition, *sdd1* mutation increased leaf stomatal density (SD) while *SDD1* over-expression led to the opposite phenotype with decreased SD. In *C. arabica*, maximum and minimum average stomatal densities were observed in full sunlight and shaded conditions respectively, providing evidence for the existence of plasticity for this characteristic in this coffee species [46, 47]. Even though no SD were observed between Rubi and IAPAR59 under moderate drought conditions [48], the *CaSLP1* expression profiles presented here do not preclude the involvement of this gene in the genetic determinism of drought tolerance in coffee.

Another interesting response concerned the differential expression of the *CaMAS1* gene encoding a putative protein containing the conserved domain [cd05326]. This domain is also found in secoisolariciresinol dehydrogenase-like proteins catalyzing the NAD-dependent conversion of (-)-secoisolariciresinol to (-)-matairesinol, like the *Arabidopsis* ABA2 protein considered to be one of the key regulators of ABA biosynthesis [49]. Based on the *CaMAS1* expression profiles presented here, it is possible that ABA synthesis was enhanced by drought in plagiotropic buds of IAPAR59 but not (or to a lesser extent) in those of Rubi. This hypothesis is also reinforced by the fact that higher *CaJAMT1* expression was observed in IAPAR59 than in Rubi buds. Indeed, in addition to well-known functions of jasmonates in plant defence mechanisms in response to biotic stress [50], recent studies also demonstrated that methyl jasmonate stimulates ABA biosynthesis under drought conditions in panicles of *Oryza sativa* [51].

Higher expression of *CaSDC1* (encoding a protein sharing 89 % similarity with the S-adenosyl-L-methionine decarboxylase from *Catharanthus roseus*) under drought conditions in IAPAR59 than in Rubi is also worth noting because this enzyme catalyzes the synthesis of polyamines (e.g. spermine, spermidine and putrescine) involved in stress tolerance in higher plants [52]. In *Theobroma cacao*, ABA and drought induced the expression of *TcSAMDC* increasing spermine and spermidine leaf contents correlated with changes in stomatal conductance [53]. More recently, *SAMDC* over-expression in transgenic rice was also shown to facilitate drought tolerance [54]. Investigation of polyamine levels in plagiotropic buds and leaves of IAPAR59 and Rubi would be of particular interest to see if these compounds are involved in drought tolerance in coffee.

In mature plants, nuclear-encoded early-light inducible proteins (ELIPs) accumulate in response to various stress conditions including ABA or desiccation [55]. These proteins are presumed to protect the chloroplast apparatus from photo-oxidation occurring after stomatal limitation of photosynthesis [56]. In a recent study, transgenic plants of *Medicago truncatula* over-expressing the *Dsp22* gene from *Craterostigma plantagineum* were shown to be able to recover from water deprivation better than wild type plants, thereby reinforcing the idea of using ELIP-encoding genes to improve abiotic stress resistance in crops [57]. Our results clearly highlight the increased expression of the *CaELIP3* (ELIP-like), *CaPSBB* (CP47-like) and *CaCAB2* (PSII Cab proteins) genes, respectively, under drought conditions. Interestingly, the expression levels of all these genes were always higher in IAPAR59 than in Rubi. These results are also in accordance with electronic Northern experiments which showed high accumulation of ELIP and Cab-encoding ESTs in cDNA libraries of *C. arabica* and *C. canephora* subjected to drought [58].

Another surprising result concerned the *CaPSBB* gene that was reverse-transcribed and detected during our qPCR experiments despite the fact that it corresponds to a chloroplast gene [59]. However, preliminary analyses of a whole genome sequence of *C. canephora* revealed the presence of a CP47/like nuclear gene [60]. Interestingly, photosystem II CP47 chlorophyll apoproteins encoding ESTs have also been reported to be expressed in *C. arabica* beans [61], leaves infected by *Hemileia vastatrix* [62] and also in the cDNA libraries (SH2 and SH3) of drought-stressed coffee plants [14, 24, 31], demonstrating increased expression of this gene under biotic and abiotic stress. As CP47 and ELIP proteins are essential for the activity and protection of the photosynthetic apparatus [55], the expression profiles reported here probably reflect a better photosynthetic and physiological status of IAPAR59 compared to Rubi.

Differential expression was also observed for the chitinase-encoding gene *CaCHI1*, with higher expression in IAPAR59 than in Rubi. An opposite situation was observed with respect to the chitinase-encoding genes *CaCHI2* and *CaCHI3*, whose expression was reduced under drought conditions. It is worth noting that the expression of these genes under drought conditions was always higher in IAPAR59 than in Rubi. These results also show that coffee chitinase-encoding genes responded in different ways to drought. A large number of chitinase-encoding ESTs were identified in the BCGP project [24], mainly in the SH2 cDNA library of drought-stressed plants of *C. arabica* var. Catuai [58], but also in the leaves of *C. arabica* infected by leaf rust [62]. Even though chitinases are defence-related enzymes induced by abiotic stress, some evidence also indicates their participation in tolerance to abiotic stress [63]. Even though the roles of pathogenesis-related proteins in abiotic stress are still not fully understood, D^T^ transgenic plants over-expressing chitinase genes have been obtained [64]. In that sense, the high level of expression for *CaCHI1* in plagiotropic buds of IAPAR59 under both control and drought conditions could have an important function in drought tolerance.

Arbutin is a phenolic glucoside (4-hydroxyphenyl-*β*-D-glucopyranoside) abundant in the leaves of many freezing- or desiccation-tolerant plants [65] and also present in coffee fruits [66]. In a previous study, down-regulation of the *CcGAS1*gene encoding arbutin synthase was reported in leaves of *C. canephora* under drought conditions [10]. The results presented here clearly demonstrated differential expression profiles for *CaGAS2* between the two cultivars of *C. arabica*. Gene expression increased under drought conditions in IAPAR59 while the opposite was observed in Rubi. Even though the presence of arbutin in coffee leaves has never been demonstrated, further analyses of this metabolite should be performed to investigate the role of this glucoside (and of other phenolic compounds) in preventing cell damage in coffee subject to abiotic stresses.

The *CaPP2* gene (encoding a putative phloem protein 2, PP2) also showed differential expression profiles, with higher expression in IAPAR59 than in Rubi. In higher plants, PP2s are sieve elements (SE) very abundant in the phloem sap. These proteins are believed to play an important role in the establishment of phloem-based defence mechanisms induced by insect attacks and feeding stress [67], but also by wounding and oxidative conditions [68]. The functions of PP2 proteins are still not clear but they could act by forming high molecular weight polymers to close (“SE plugging”) the sieve pores caused by external injuries mainly due to biotic stress [69]. When *Arabidopsis* was treated with HrpN_Ea_ (a proteinaceous elicitor of plant defences produced by gram-negative plant pathogenic bacteria), the suppression of phloem-feeding activities by aphids was attributed to over-expression of the PP2-encoding gene *AtPP2-A1* [70]. Other studies showed that HrpN activated ABA signalling, thereby inducing drought tolerance in *Arabidopsis thaliana* [71]. Based on these results, the involvement of PP2 proteins in plant response mechanisms to abiotic stress can be hypothesized, for example by maintaining (or protecting) the integrity of vessels under drought conditions by forming sieve plate filaments upon oxidation [72]. In that case, higher synthesis of CaPP2 which would be expected to occur in IAPAR59 plagiotropic buds under drought conditions could play a role in drought-tolerance by reducing sap-flow in young leaves and consequently increasing the water use efficiency of this cultivar [48].

Other interesting results concerned the gene expression stability of the *CaAEP1* (putative aldose 1-epimerase) and *CaIPS1* (myo-inositol 1-phosphate synthase) genes observed in IAPAR59 under control and drought conditions, whereas expression of both genes decreased under drought conditions in Rubi. Plant cells use myo-inositol to synthesize a variety of low molecular weight compounds and sugar alcohols such as the galactinol, a key element in the formation of raffinose family oligosaccharides. Nishizawa et al. [73] found that plants with high galactinol and raffinose contents were less susceptible to oxidative stress. In *C. arabica*, up-regulation of *CaGolS* genes involved in galactinol biosynthesis was reported in leaves of plants subjected to severe drought [74]. In addition, drought up-regulated the expression of mannose 6-phosphate reductase (involved in mannitol biosynthesis) in leaves of *C. canephora* [10, 11] and *C. arabica* [75, 76]. Even though little is known about the biochemical mechanisms of drought tolerance in coffee, the accumulation of carbohydrates expected in leaves of drought-stressed plants as a consequence of the up-regulated expression of these genes, could play an important role in the genetic determinism of this phenotype in coffee [77].

In addition to the previously described genes, our results also identified several orphan genes that presented differential expression profiles between the cultivars and treatments, such as *CaUNK2*, *CaUNK3* and *CaUNK4* whose expression was highly induced under drought conditions in IAPAR59 and to a lesser extent in Rubi. Orphan genes are also expected to interact specifically with the environment as a consequence of lineage-specific adaptations to that environment [78].

Interestingly, the expression profiles of the *CaUNK2* and *CaUNK3* orphan genes were very similar to those of Type II nsLTP-encoding genes, with high expression mainly detected under drought conditions in plagiotropic buds of IAPAR59 but not in those of Rubi. Up-regulation of *LTP* genes under drought conditions is well documented in higher plants [79–81]. Lipid transfer proteins (LTPs) are thought to be involved in the transfer of lipids through the extracellular matrix for the formation of cuticular wax [82]. In fact, together with the lipophilic cutin polymer matrix, waxes enter in the composition of cuticle, which forms the first barrier between plants and environmental stresses by limiting non-stomatal water loss and gas exchanges, hence mitigating the effects of drought by controlling water loss associated with epidermal conductance [83]. In *Nicotiana glauca,* LTP genes are predominantly expressed in the guard and epidermal cells and are induced under drought conditions [84], providing evidence that LTP play an important role in the development of drought tolerance. Even though the up-regulation of *CaLTP* genes observed under drought in plagiotropic buds of IAPAR59 cannot explain directly the greater thickness of leaf cuticle observed in this cultivar than in Rubi, these results strongly suggested that lipid metabolism plays a major role in coffee drought tolerance.

As reported in other higher plants, our study also highlighted the differential expression of many genes encoding proteins known to be over-expressed under biotic stress (e.g. chitinases and PP2), by drought. The fact that our experiment was conducted with drought-stressed plants grown under uncontrolled (field) conditions, could explain such a situation. However, it is also probable that these results reflect a biological reality since it is well known that crosstalk exists in higher plants between signalling pathways for biotic and abiotic stress responses [85].

## Conclusions

During this work, we produced some new transcriptomic information for *C. arabica* with a total of 34.7 Mbp of sequences assembled into 43,087 clusters (41,512 contigs and 1,575 singletons) from genes expressed in plagiotropic shoot apices enriched in meristems and primordium leaves in D^T^ (IAPAR59) and D^S^ (Rubi) cultivars grown under control and drought conditions. Major differences between these plants concerned their phenotypic behaviour (e.g. predawn leaf water potential, *Ψ*_pd_) and transcriptome expression profiles. Differences between these plants affected genes of specific pathways such as those involved in abscisic acid biosynthesis, perception and transduction of drought stress, plant development and lipid metabolism. In that sense, the present study increased the number of CGs potentially involved in the genetic determinism of drought tolerance firstly identified in *C. canephora*. Because *C. arabica* is an amphidiploid species (originating from a natural hybridization event between *C. canephora* and *C. eugenioides*), its transcriptome is a mixture of homologous genes expressed from these two sub-genomes in which *C. eugenioides* is assumed to express genes mainly for proteins involved in basal biological processes (e.g. photosynthesis), while the *C. canephora* sub-genome is assumed to regulate Arabica gene expression by expressing genes for regulatory proteins and adaptation processes [86]. In this genetic context, it is possible that the characteristics of IAPAR59 that enable it to better withstand drought stress than Rubi, really originated from the specific expression of *C. canephora* genes recently introgressed (through the Timor hybrid HT832/2 [19]) in this cultivar of *C. arabica* [33]. Even though this study provides further indications about the way in which different coffee cultivars activate their transcriptomes, additional work is still required to understand how epigenetics and epistasis regulate gene expression in the different coffee sub-genomes (*CaCe* and *CaCc*) in *C. arabica* under drought conditions.

### Source of the plant materials and permissions

This work was carried out as part of the scientific cooperation project entitled “Study of genetic determinism of drought tolerance in coffee” (2006–2010) approved between Embrapa and CIRAD. It complied with all institutional, national, or international guidelines. In the frame of this project, field experiments were conducted at the Cerrado Agricultural Research Center (Planaltina-DF, Brazil) with all permissions of partners and in accordance with local legislation.

### Ethics approval and consent to participate

Not applicable.

### Consent to publish

Not applicable.

### Availability of supporting data

The reads were submitted to GenBank and to the BioProject/NCBI database under the accession number PRJNA282394.

## Additional files

Additional file 1: Table S1. Summary of Blast2GO automatic annotation of the transcriptome clusters. (XLS 15226 kb) Additional file 2: Figure S1. Complete bioinformatics pipeline of the transcriptome assembly and automatic annotation methods used in this work. (TIF 105 kb) Additional file 3: Table S2. Summary of the DEseq/ EdgeR fold changes and *p*-values between the cultivars (I59: IAPAR59 and RUB: Rubi) and between control (C) and drought (D) conditions. These tables also contain Blast2GO automatic annotation of the transcriptome clusters. Table lanes coloured in grey are related to clusters aligned to the new candidate genes tested by RT-qPCR (see Table 1). (XLS 51895 kb)

## Abbreviations

EST: expressed sequence tag
qPCR: quantitative polymerase chain reaction
