# Supplementary figures and images for: Identification of candidate genes for drought tolerance in coffee by high-throughput sequencing in the shoot apex of different Coffea arabica cultivars
Source: BMC Plant Biol. 2016 Apr 19;16:94. doi: 10.1186/s12870-016-0777-5 (PMC4837521; doi:10.1186/s12870-016-0777-5)

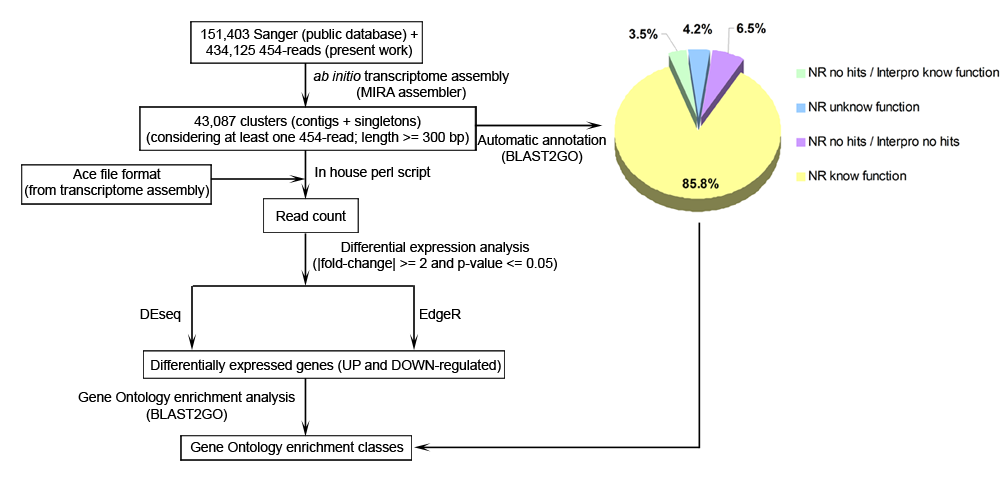

Supplement: Additional file 2: Figure S1. — Complete bioinformatics pipeline of the transcriptome assembly and automatic annotation methods used in this work. (TIF 105 kb) [file 12870_2016_777_MOESM2_ESM.tif]
